# Burden, coping strategies, and family caregivers of older people with clinical frailty in the COVID-19 context

**DOI:** 10.1590/0034-7167-2024-0324

**Published:** 2025-11-21

**Authors:** Luana Vitro, Maria Goreti da Silva Cruz, Ana Lúcia de Moraes Horta

**Affiliations:** I Universidade Federal de São Paulo. São Paulo, São Paulo, Brazil

**Keywords:** Caregivers, Caregiver Burden, Coping Skills, Aged, Frailty., Cuidadores, Carga del Cuidador, Habilidades de Afrontamiento, Anciano, Fragilidad.

## Abstract

**Objectives:**

to assess the burden and understand the coping strategies of family caregivers of older people with clinical frailty in the household.

**Methods:**

a mixed-method cross-sectional study with a two-stage explanatory sequential strategy was conducted. In the quantitative stage, 170 participants answered the socio-contextual questionnaire, EMEP and QASCI. In the qualitative stage, 31 participants answered a semi-structured interview, using Bardin’s framework.

**Results:**

sample profile: 82% women aged 50-69 (65.9%). 56.5% had family help with care and 57.6% had no professional caregiver. The 31 interviews generated three categories: “caregiver burden”, “coping strategies”, and “support network to promote care”.

**Conclusions:**

caring for older people with clinical frailty triggers changes in family structure and dynamics, causing burden, which can be reduced with an effective family and professional support network as a coping strategy.

## RESUMEN

**Objetivos:**

evaluar la carga y comprender las estrategias de afrontamiento de los cuidadores familiares de ancianos con fragilidad clínica en el domicilio.

**Métodos:**

estudio transversal con un método mixto, estrategia secuencial explicativa en dos etapas. En la etapa cuantitativa, 170 participantes respondieron al cuestionario socio-contextual, EMEP y QASCI. En la etapa cualitativa, 31 participantes respondieron a una entrevista semiestructurada, basada en el marco de Bardin.

**Resultados:**

perfil de la muestra: 82% mujeres de 50-69 años (65,9%). El 56,5% contaba con ayuda familiar para el cuidado y el 57,6% no tenía cuidador profesional. Las 31 entrevistas generaron tres categorías: “carga del cuidador”, “estrategias de afrontamiento” y “red de apoyo para promover el cuidado”.

**Conclusiones:**

el cuidado de una persona mayor con fragilidad clínica desencadena cambios en la estructura y dinámica familiar, causando sobrecarga, que puede reducirse con una red de apoyo familiar y profesional eficaz como estrategia de afrontamiento.

## INTRODUCTION

The increase in life expectancy around the world has caused social and family changes in all societies. In Brazil, it has risen to 76.2 years, and in the world to 73.4 years^(1)^. The United Nations (UN) classifies older people as: older adult (55 to 64 years old), young-old (65 to 74 years old), middle-old (75 to 80 years old), and oldest-old (81 years old and over)^(2)^.

The UN classifies older people based on the level of development of each country. In developed countries, such as Europe, older people are considered to be aged 65 or over, while in Brazil older people are considered to be aged 60 or over^(3)^.

As people get older, they are more susceptible to health complications, and clinical frailty is one of the major geriatric syndromes that affect the older population and has an impact on the families^(4)^. As a care system, families can be impacted because they are the people responsible for caring for older people, according to the Statute of the Elderly, which regulates the rights guaranteed to people aged 60 or over^(5)^.

As a result, a family member is chosen to be the main caregiver of the dependent older person, whether they are prepared or not^(6)^. In Brazil, studies show an increase in the number of older adults and young-old people who are caregivers^(7)^.

The care routine can affect family members’ health, given the increase in workload and the demand for physical and emotional effort^(8)^. The main family caregiver needs to adapt to a new routine, in which they have to provide basic care, help with activities of daily living (ADLs), and health care, in addition to managing the support network, where it exists, and the household’s problems.

Other significant consequences can arise in the life of the main family caregiver, the main one being burden^(9)^. Burden can be defined as an individual perception measured from the personal analysis of the impacts caused by the act of caring, with emotional, physical, and financial consequences^(10)^.

Coping strategies can be defined as efforts made to manage problematic situations. They are classified as problem-focused (in which actions are taken to solve problems) and emotion-focused (in which emotions and internal demands are managed)^(11)^.

Having an effective support network is a coping strategy adopted by some main family caregivers, but for many families the support network is absent or ineffective. Studies show that the presence of a support network can relieve stress and help improve the health of family caregivers^(9)^.

The support network can be classified into formal and informal. The former is made up of family members and acquaintances. The informal network is made up of health professionals and institutions capable of providing help and technical guidance^(12)^.

The nursing team is an important formal support network for family caregivers of older people, as they are able to provide technical and practical guidance^(13)^. However, in addition to this guidance and assistance in the practice of care, it is necessary to expand the team’s attention to the family.

Studies on this subject are still needed so that the nursing team can adopt a systemic approach to care and include the patient’s family in their professional practice.

In view of the above, this study sought to answer the questions: “What was the experience of caring for older people with clinical frailty in their households in the context of the COVID-19 pandemic like, from the perspective of family caregivers?” and “What coping strategies were used during the COVID-19 pandemic?”.

## OBJECTIVES

To assess the burden and understand the coping strategies of family caregivers of older people with clinical frailty in the household.

## METHODS

### Ethical aspects

The study followed ethical precepts and was approved by the UNIFESP ethics committee. The participants were informed about the objectives of the research by means of a Free and Informed Consent Form.

### Study design, period and location

This is a mixed-method cross-sectional study with an explanatory sequential strategy, the first quantitative stage being an observational study in epidemiology guided by the STROBE tool; and the second qualitative stage being guided by the COREQ checklist^(14-16)^. It was carried out in two stages in different municipalities in the state of São Paulo from July 2022 to April 2023^(14)^. In the quantitative stage, data were collected online using Google Forms. In the qualitative stage, data were obtained through interviews on the Google Meet platform and in person at the Center for Nursing Care and Education at the Federal University of São Paulo (CAENF-UNIFESP).

### Sample, inclusion and exclusion criteria

The sample consisted of 170 main family caregivers over the age of 18 who lived in the same household during the COVID-19 pandemic, in accordance with the sample calculation. Subsequently, all participants were randomly invited, with 31 family members agreeing to participate in the qualitative stage based on their availability of time.

The study included family members of elderly individuals with clinical frailty who lived in the same household and were over 18 years old. This study considered elderly individuals with clinical frailty aged 65 and older who had one or more characteristic symptoms of the syndrome. Participants who did not meet these criteria were excluded.

### Study protocol

In the quantitative stage, the researchers prepared and applied a Socio-Contextual Questionnaire, in addition to the following instruments validated in Brazil: the Ways of Coping Questionnaire (EMEP), to measure coping strategies in relation to specific stressors, and the Informal Caregiver Overload Assessment Questionnaire (QASCI), to measure the physical, emotional, and social burden of informal caregivers^(17,18)^.

The data for the qualitative stage was obtained from semi-structured interviews carried out by researchers with expertise in the subject. Before taking part, family members were informed about the study and their doubts were clarified. Each interview was audio-recorded with the consent of the family member and lasted one hour, beginning with the following guiding questions: “What was the experience of caring for your older family member with clinical frailty like?”, “What coping strategies were used in caring for them during the COVID-19 pandemic?”.

### Analysis of results and statistics

The Statistical Package for the Social Sciences, version 21.0, was used for the statistical analysis. Spearman’s correlation coefficient was used to correlate the scores of the EMEP domains with the QASCI domains. The Mann-Whitney test and the Kruskal-Wallis test were used to compare the scores of the EMEP domains and the QASCI domains with the categorical social and contextual variables, with a significance level of 5% (p-value < 0.05). The sample size calculation revealed an n of 170 for the comparison between the EMEP domains and the presence of dementia in older people, the “Sample Size for Difference Between Means” was used, with a significance level of 5% and a test power of 80%.

The information obtained was subjected to content analysis using the MAXQDA.2024 software, based on the thematic analysis proposed by Bardin^(19)^. The themes were selected and part of the narratives were coded to create categories. After this stage, axial and selective coding was carried out, resulting in the following categories: “Burden generated by the care provided”, “Coping strategies in the face of the care situation”, and “Support network to promote care”.

## RESULTS

Table 1 shows the characteristics of the sample with socio-contextual data, made up of 170 participants, 82% of whom were women aged 50-69 (65.9%), 40% employed and 25.9% retired, 68% with children, 31% with an income between R$ 4,801 - 7,200.00. When asked about coping, 15.3% reported doing nothing to cope, while 15.3% reported acceptance and 13.5% seeking knowledge. When asked about other ways of dealing with the demands of care, 32.9% did not use other ways and 14.7% said faith and gratitude.

**Table 1:** Characteristics of the socio-contextual variables of family caregivers, Brazil, May-December/2022

Variables	Total
**Age group**	
18-29	2 (1.2%)
30-49	44 (25.9%)
50-69	112 (65.9%)
70 or older	12 (7.1%)
Total number of participants	170
**Sex**	
Female	140 (82.8%)
Male	29 (17.2%)
Total number of participants	169
Did not answer	*1*
**How many people live in your household?**	
2 people	5 (2.9%)
3-4 people	119 (70%)
5 or more people	46(40.6%)
Total number of participants	170
**Do you have children?**	
Yes	117 (68.8%)
No	53 (31.2%)
Total number of participants	170

*Descriptive analysis of categorical variables. Frequency and percentage calculation.*

Regarding the data on the older people receiving care, Table 2 shows that the age range was 75-79 years (27.1%) and 80-84 years (22.9%), 34.1% had 5-6 symptoms and 24.1% had 7-8 symptoms which classified them as clinically frail, 43.5% had lived with the family for more than five years and 22.9% for three years.

**Table 2:** Characteristics of socio-contextual variables related to older people with clinical frailty, Brazil, May-December/2022

Variables	Total
**What is the age of the older person under your care?**	
65-69	7 (4.1%)
70-74	17 (10%)
75-79	46 (27.1%)
80-84	39 (22.9%)
85-89	33 (19.4%)
90-94	22 (12.9%)
95-99	5 (2.9%)
Over 100	1 (0.6%)
Total number of participants	170
**What are its signs and symptoms?**	
1-2 symptoms	18 (10.6%)
3-4 symptoms	29 (17.1%)
5-6 symptoms	58 (34.1%)
7-8 symptoms	41 (24.1%)
9-10 symptoms	24 (14.1%)
Total number of participants	170
**How long has the older person lived with you (in years)?**	
1	6 (3.5%)
2	19 (11.2%)
3	39 (22.9%)
4	32 (18.8%)
5	74 (43.5%)
Total number of participants	170
**Does any family member help you with the care?**	
Yes	96 (56.5%)
No	74 (43.5%)
Total number of participants	170
**How are you related?**	
Sibling/Spouse	54 (31.7%)
Parent	20 (11.8%)
Child/grandchild	26 (15.3%)
Does not receive help	70 (41.2%)
Total number of participants	170
**Do you have help from a professional caregiver?**	
Yes	72 (42.4%)
No	98 (57.6%)
Total number of participants	170

*Descriptive analysis of categorical variables. Frequency and percentage calculation.*

When asked about their support network, 56.5% of the participants reported having family help with care, 31.7% being helped by siblings/spouses and 15.3% by children/grandchildren, whereas 57.6% did not have help from a professional caregiver.

Analysis of the scales showed that emotional and financial burden is present in family caregivers of older people diagnosed with stroke, Parkinson’s, and dementia, even if they have family support.

When burden was correlated with coping strategies, according to Table 3, it was noted that the greater the emotional burden and the implications for their personal life caused by providing care without family help, the greater the search for religious practices as a coping strategy for the main family caregiver. However, there is also a greater search for religious practices by family caregivers who have a professional support network, when compared to family caregivers without a professional support network.

**Table 3:** Characteristics of the variables of correlation of Ways of Coping Questionnaire domains by Informal Caregiver Overload Assessment Questionnaire domains, Brazil, May-December/2022

		Problem-Focused Coping	Emotion-Focused Coping	Search for Religious Practices/Fantastical Thinking	Search for Social Support
**Emotional Burden**	R	0.07	0.23	0.21	0.01
	*p* value	0.3919	**0.0026**	**0.0062**	0.8627
	N	170	170	170	170
**Implications for the caregiver's personal life**	R	0.05	0.20	0.19	-0.04
	*p* value	0.5572	**0.0099**	**0.0111**	0.5812
	N	170	170	170	170
**Financial burden**	R	-0.03	0.00	0.08	-0.04
	*p* value	0.6951	0.9659	0.2846	0.5834
	N	170	170	170	170
**Reactions to demands**	R	0.04	0.13	0.02	0.01
	*p* value	0.6005	0.0987	0.8337	0.9349
	N	170	170	170	170
**Efficacy and control mechanisms**	R	-0.04	0.00	-0.13	-0.13
	*p* value	0.5764	0.9489	0.1016	0.0979
	N	170	170	170	170
**Family support**	R	0.00	0.18	0.12	-0.11
	*p* value	0.9988	**0.0184**	0.1321	0.1470
	N	170	170	170	170
**Satisfaction with role and family member**	R	-0.18	-0.05	-0.12	-0.10
	*p* value	**0.0223**	0.5513	0.1090	0.2070
	N	170	170	170	170

*R - Spearman’s Correlation Coefficient.*

The main family caregiver who has family support and greater implications for their personal life shows greater coping focused on emotion. While the main family caregiver who has help from their spouse and no professional support network has less problem-focused coping.

Family members who live with one or two people in the same household without a family support network experience greater implications in their personal lives, compared to family members who have help from siblings or spouses, as well as having a greater problem-focused coping.

Family members who have lived with the older people for five years or more and are aged between 50-69 have less family support, whereas family members who have lived with the older people for three or four years and are aged between 40-49 have family support.

By combining the codes from the 31 interviews, the textual and theoretical content analysis of the narratives generated three categories and sub-themes that responded to the proposed objectives.

**Figure 1:**
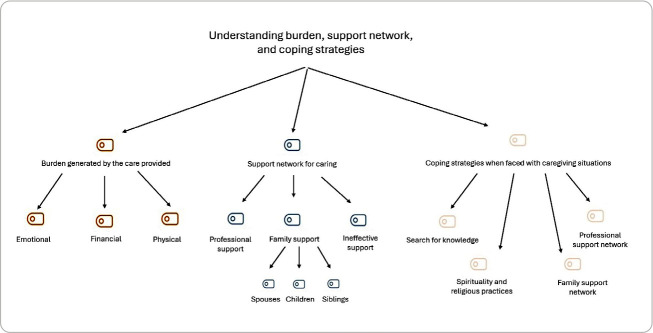
Map of categories and sub-themes, São Paulo, São Paulo, Brazil, 2023

Starting from the categorization of the textual analysis to check for possible approximations and similarities between the categories and sub-themes of the open coding, the MaxMaps tool of the MAXQDA.2024 software was used (Figure 1).

After the initial open coding, the categories and sub-themes relating to the experiences of the evaluative focus group were observed. The first category refers to family members’ perception of the burden generated by the care provided, as exemplified by the following statements.

### Category 1: Burden generated by the care provided

In the first category, the narratives of some family members stand out, with reports of an ineffective family and professional support network, causing increased burden, mainly related to the COVID-19 pandemic.

*I feel very overwhelmed looking after him, even though I have help* [...] *I feel very tired!* (E.3)

*I’ve felt very overwhelmed, emotionally and physically, in every way, since the pandemic made things more difficult.* (E.8)

*After the pandemic, I felt the emotional burden, I think it all added up! My wife and I are overwhelmed emotionally, physically, and financially, we have no one to rely on apart from the caregiver.* (E.27)

The presence of burden implied a worsening of the family caregiver’s physical health and mental health, highlighted in the following statements.

*I began to feel pain all over my body, in my joints, my back, everywhere!* (E.1)

*To tell you the truth, I was even more tired and stressed out!* (E.20)

Another highlight is the presence of burden related to the reversal of roles in the family system, especially when they began to care for their parents, which is reminiscent of anticipatory mourning.

*I felt emotionally overwhelmed because I was saddened by her* [mother’s] *situation, I understand that I went through and am going through the mourning of an independent mother.* (E.16)

*At many times I felt very emotionally overwhelmed, seeing my mother in need of basic care really touched me, I didn’t expect to have to go through this with her* [mother]. (E.19)

*It’s a weight, an indescribable burden, only those who go through it really know what it’s like to have to see your parents like this and still have to take care of them, to learn how to take care of them. At the end of the day I feel exhausted, physically and emotionally.* (E.30)

The second category refers to the coping strategies developed in the face of the care situation. The following narratives exemplify some of the strategies developed.

### Category 2: Coping strategies when faced with caregiving

In the second category, family members reported having developed emotionand problem-focused strategies.

*I also cope by detaching myself from the situation, as if they weren’t my mother and father, as if they were other people I was looking after. Sometimes I detach myself, so it doesn’t get so heavy!* (E.7)

*Getting everything he’s entitled to under the health plan, taking them to the doctors, keeping an eye on the caregivers who come to the house, these are things that give me a lot of security and peace of mind... and knowing that I’m doing everything I can.* (E.25)

*After I took her* [mother] *to the doctor, things started to change for me, I began to understand what my mother really had and I changed the way I dealt with the situation, I talked a lot with my wife too.* (E.27)

Religious practices, spirituality, and the search for knowledge were important coping strategies, according to family members’ reports.

*I haven’t stopped praying, it strengthens me a lot, even though I don’t go to church anymore.* (E.18)

*I asked God a lot to give me patience, because I have very little patience.* (E.21)

*I was looking for knowledge so that I could understand what was happening and so that I could look after my mother in the best possible way.* (E.9)

The third category refers to the support network for exercising care and elucidates the characteristics of the network such as family support, professional support, and ineffective support, as exemplified in the following statements.

### Category 3: Support network to promote care

The third category shows family caregivers who only have a professional or institutional support network, complaining that they cannot count on an effective family support network.

*My support network is only the Home Care Technicians and my children financially when I ask, but they don’t help me enough. I would like them to be more involved, to come home more.* (E.3)

*Very rarely did my brother come to help me when I asked him to. The Life Group helps me a lot, the caregivers help me.* (E.5)

*As a support network we only have the caregiver, the family doesn’t help us at all, not even in taking turns or looking after us.* (E.6)

Regarding care demands, the participants mostly identified their spouses and siblings as their family support network.

*My sisters helped me a lot in this whole process, we are four sisters and all married, all the families helped each other a lot to take care of her* [mother]*, she* [mother] *was our main focus!* (E.10)

*My husband is the only person who helps me look after my brother when he’s at home.* (E.17)

*My support network has always been my brother.* (E.19)

*My sister is the only person who helps me take care of him* [father]*. The rest of my family, my other siblings and relatives, don’t help me at all, they don’t even show up at my house, they don’t call me.* (E12)

## DISCUSSION

The world’s ageing population is part of sociodemographic changes. In this scenario, longer life expectancy leads to the development of clinical frailty and non-communicable chronic diseases (NCDs) in older people, triggering a greater need for care. These factors increase the demand for care and, consequently, the emotional, physical, and financial burden on family caregivers, especially women^(20,21)^.

It was shown that the majority of family caregivers are women (82.8%) who are older adults and young-old adults aged 50-69 (65.9%) who have less family support when compared to family caregivers aged 30-49. These data corroborate a study that characterized the profile of caregivers of older people and proves the phenomenon of “feminization of care”, i.e. women as caregivers^(22)^.

In addition to the cultural issue of family care provided by women, another important factor is the premature death of men, which according to Ministry of Health indicators occurs between the ages of 30 and 69 due to NCDs, violence, and other causes. This data is worrying, as in 2023 alone, Brazil recorded 111,544 deaths caused by NCDs, 55.7% of which were men and 44.3% women. This scenario aggravates the burden and accumulation of women’s roles within the family system, and also affects the quality of care provided^(23)^.

The qualitative results of this study confirm the “feminization of care”. It was possible to identify in the narratives of family caregivers the scarce participation of men in the care provided to older people with clinical frailty, corroborating Brazilian qualitative research which observed the full dedication of men to the care of an elderly relative while they are single, but once they start a relationship with a woman, she starts to take responsibility for family care^(24)^. As family caregivers advance in the condition of older adults and young-old adults, they can develop an accumulation of roles and functions, a factor that can aggravate their health.

In this study, it was identified that caregivers participating in the context of the COVID-19 pandemic had their burden intensified, due to the worsening health of older people, mostly those aged 75-84, with clinical fragility under their care, presenting five to eight classificatory symptoms, added to the diagnoses of stroke, Parkinson’s, dementias, and other NCDs. This is explained by the deterioration in health care in the public network and a reduction in the provision of care in the private network, due to the need for social isolation.

These findings are in line with research that concluded that older people with or without dementia had clinical changes during the pandemic due to social isolation, forced physical inactivity, and restriction of external stimuli, also presenting cognitive impairment^(25,26)^. In this respect, the worsening health of dependent older people intensified the demand and burden of care, implying an increase in physical, emotional, and financial burden, because, in addition to pandemic isolation, these caregivers also had poor health monitoring.

The data obtained shows less family support for care when the older people have lived in the same household for five years or more, compared to three or four years when there is more support. In line with this, a cross-sectional study carried out with 348 older people who live with their families showed that as the years go by and care dependency progresses, there is a reduction in the family support network for family caregivers. In this scenario, it is clear that nursing is needed as a professional support for dependent older people and family caregivers^(27)^.

Other aspects highlighted in the caregivers’ narratives were the implications and/or repercussions on their personal lives, such as worsening physical health. Some reported back problems, fatigue, and mental health problems, such as stress and anxiety related to the lack of a family support network. These factors were also found in a study carried out under the coordination of the Oswaldo Cruz Foundation, which showed that living with an older person who was dependent on care during the pandemic led to a deterioration in health, mood, and sleep^(28)^. It should be noted that the worsening health of family caregivers can have an impact on the condition of the care provided. In order to minimize this situation, it is necessary for the health team to extend their care to the families of older people.

Faced with the burden of care, family caregivers develop strategies focused on the emotion or the problem. This study highlights the search for religious practices and spirituality as an important strategy for overcoming adversity, as well as the search for knowledge through reading and discussion with health professionals and virtual social networks.

In line with this, a mixed-method study that investigated spirituality/religiosity as a coping strategy in the context of the COVID-19 pandemic concluded that spirituality/religiosity, when stimulated, contributes to minimizing adverse damage to mental health^(29)^. It can be seen that the spiritual dimension is able to contribute to improving physical and emotional well-being, but it should be noted that other strategies such as social interaction, physical activity and leisure time, seeking specialized care, individual and family therapy, and integrative and complementary therapies, are essential for the self-care of family caregivers^(30)^.

Regarding coping strategies and care, family and professional support are crucial in promoting health and well-being. The qualitative data reported an increase in the burden on caregivers who are older adults and young-old adults due to the ineffectiveness of the family support network (mostly made up of siblings and spouses), even in situations where there is a professional support network. In line with this, a qualitative study concluded that caregivers who were older people had different support networks that were effective in minimizing the burden of care provided^(31)^. It can therefore be seen that an effective support network is a coping strategy that reduces the burden, among which the help of the nursing team in promoting extended care for the family stands out.

Nursing as part of the professional support network can provide health education and plan care for the family, according to the demands of older people with clinical frailty and the main family caregiver. In the interviews in this study, the importance of the nursing team’s professional support network based on systemic theory became clear.

The Systems Theory encourages nursing to promote comprehensive, systemic care that includes older people and their main family caregiver, considering health to be a dynamic process. From this perspective, the health team, including the nursing team, needs to broaden its patient-centered approach to the family, help the family to identify and develop a support network, and collaborate in developing coping strategies^(32,33)^. In addition, nursing can help promote autonomy in the functioning of the family system, helping to identify its strengths and develop its own resources. Quantitative research carried out in a city in the state of Minas Gerais corroborates the study in question by concluding that nursing helps to support family health^(34)^.

### Study limitations

A limitation of this study is that it is only a regional study for the state of São Paulo, with a number of participants that does not represent the entire region or country. Data were collected in the context of the COVID-19 pandemic, which is an obstacle to greater comprehensiveness. It should be noted that although the current scenario is post-pandemic, families continue to be overburdened by the demands of care, which have been exacerbated. In view of the above, it is important to invest in new research to propose and evaluate interventions to alleviate the burden and help develop strategies for family caregivers who are older adults and young-old adults in caring for dependent older people.

### Contributions to the field of Nursing

This study makes significant contributions to the field of nursing, highlighting the importance of expanding care for the families of older people, in other words, expanding care strategies for families. In view of this, it becomes relevant and urgent to establish care action planning and personalized interventions for families according to their specific demands, since an older adult or young-old adult caring for other older people also needs health care.

## CONCLUSIONS

The phenomenon of having an older person with clinical frailty in the household has increased worldwide, generating changes in the structure and dynamics of the family, especially for the main family caregiver who is an older or young-old adult. This study shows that the increased burden on family caregivers has an impact on the care routine, with the family and social support network being an effective coping strategy, as well as the search for religious practices, spirituality, and knowledge through reading and discussion with health professionals and virtual social networks.

It is important to pay attention to the health of family caregivers who are older people, who can become ill as a result of the burden added to their clinical demands. In view of this, it is essential that health teams expand their work to include older people’s families, in line with objective 3, item 3.4 of the United Nation’s 2030 Agenda of Sustainable Development Goals (SDGs).

## Data Availability

The research data are available within the article.
