# Supplementary material for: Burden, coping strategies, and family caregivers of older people with clinical frailty in the COVID-19 context
Source: Rev Bras Enferm. 2025 Nov 21;78(Suppl 2):e20240324. doi: 10.1590/0034-7167-2024-0324 (PMC12659913; doi:10.1590/0034-7167-2024-0324)
Supplement: Translation

# Translation: Sobrecarga, estratégias de enfrentamento e familiares cuidadores de idosos com fragilidade clínica no contexto COVID-19

**DOI:** 10.1590/0034-7167-2024-0324pt

**Authors:** Luana Vitro, Maria Goreti da Silva Cruz, Ana Lúcia de Moraes Horta

**Affiliations:** I Universidade Federal de São Paulo. São Paulo, São Paulo, Brasil

**Keywords:** Familiares Cuidadores, Sobrecarga do Cuidador, Estratégias de Enfrentamento, Pessoa Idosa, Fragilidade.

## Abstract

**Objetivos:**

avaliar a sobrecarga e compreender as estratégias de enfrentamento de familiares cuidadores de pessoas idosas com fragilidade clínica no domicílio.

**Métodos:**

estudo transversal de método misto, estratégia sequencial explanatória em duas etapas. Na etapa quantitativa, 170 participantes responderam ao questionário sociocontextual, EMEP e QASCI. Na etapa qualitativa, 31 participantes responderam a uma entrevista semiestruturada, com referencial de Bardin.

**Resultados:**

perfil da amostra: 82% mulheres de 50 a 69 anos (65,9%). 56,5% têm ajuda familiar no cuidado, e 57,6% não têm ajuda de cuidador profissional. As 31 entrevistas geraram três categorias: “sobrecarga gerada pelo cuidado prestado”, “estratégias de enfrentamento diante da situação de cuidar” e “rede de apoio para promover o cuidado”.

**Conclusões:**

cuidar de uma pessoa idosa com fragilidade clínica desencadeia modificações na estrutura e dinâmica familiar, ocasionando a sobrecarga, que pode ser reduzida com a rede de apoio familiar e profissional eficaz como estratégia de enfrentamento.

## INTRODUÇÃO

O avanço da expectativa de vida no mundo tem causado mudanças sociais e familiares em todas as sociedades, no Brasil, elevou-se para 76,2 anos e, no mundo, para 73,4 anos^(1)^. A Organização das Nações Unidas (ONU) classifica as pessoas idosas em: pré-idosos (de 55 a 64 anos), idosos jovens (de 65 a 74 anos), idosos velhos (de 75 a 80 anos) e idosos mais velhos (a partir de 81 anos)^(2)^.

A ONU classifica a pessoa idosa com base no nível de desenvolvimento de cada país, sendo nos países desenvolvidos, como os europeus, consideradas pessoas idosas aquelas com 65 anos ou mais, no Brasil considera-se a pessoa idosa a partir dos 60 anos^(3)^.

Com o envelhecimento, há maior suscetibilidade a complicações de saúde, sendo a fragilidade clínica uma das importantes síndromes geriátricas que acometem a população idosa e gera impactos na família^(4)^. A família como sistema de cuidado pode ser impactada porque é uma das responsáveis pelo cuidado da pessoa idosa de acordo com o estatuto da pessoa idosa, que regula os direitos assegurados às pessoas com idade igual ou superior a 60 anos^(5)^.

Dessa forma, elege-se ou é eleito um familiar cuidador principal para ser o responsável pelos cuidados prestados à pessoa idosa dependente, estando ele preparado ou despreparado^(6)^. No cenário brasileiro, estudos evidenciam que cresce o número de pré-idosos e idosos jovens cuidadores^(7)^.

A rotina de cuidados pode acometer a saúde do familiar, visto o aumento da carga de trabalho e da demanda de esforços físicos e emocionais^(8)^. Esse familiar cuidador principal precisa adaptar-se a uma nova rotina em que passa a realizar os cuidados básicos, auxiliar nas Atividades de Vida Diária (AVDs) e na atenção em saúde, além de administrar a rede de apoio, quando existente, e as pendências da casa.

Outras significativas consequências podem surgir na vida do familiar cuidador principal, sendo a sobrecarga a principal delas^(9)^. A sobrecarga pode ser definida como uma percepção individual mensurada a partir da análise pessoal dos impactos causados pelo ato de cuidar, com consequências emocional, física e financeira^(10)^.

As estratégias de enfrentamento podem ser definidas como esforços desenvolvidos para gerenciar situações problemáticas. São classificadas em: focadas no problema (em que há o desenvolvimento das ações para solução de problemas) e focadas na emoção (em que há o desenvolvimento de manejo de emoções e demandas internas)^(11)^.

Possuir rede de apoio eficaz é uma estratégia de enfrentamento adotada por alguns familiares cuidadores principais, porém, para muitas famílias, a rede de apoio é ausente ou ineficaz. Estudos mostram que a presença de rede de apoio pode aliviar o estresse e auxiliar na melhora da saúde do familiar cuidador^(9)^.

A rede de apoio pode ser classificada em formal e informal. Esta é composta por familiares e conhecidos. Enquanto aquela é composta por profissionais e instituições de saúde capazes de promover auxílio e orientação técnica^(12)^.

A equipe de enfermagem é uma importante rede de apoio formal para os familiares cuidadores de pessoas idosas, visto que são capazes de fornecer orientações técnicas e práticas^(13)^. Porém, além dessas orientações e auxílio na prática de cuidar, é necessária a ampliação da atenção da equipe para a família.

Dessa forma, ainda são necessários estudos sobre a temática para que a equipe de enfermagem adote sua atenção na perspectiva sistêmica e inclua a família do paciente em sua prática profissional.

Perante o exposto, este estudo buscou responder aos questionamentos: “Como foi a experiência no cuidado prestado à pessoa idosa com fragilidade clínica em seu domicílio, no contexto da pandemia da COVID-19, a partir da perspectiva do familiar cuidador?” e “Quais as estratégias de enfrentamento utilizadas no cuidado durante a pandemia COVID-19?”.

## OBJETIVOS

Avaliar a sobrecarga e compreender as estratégias de enfrentamento de familiares cuidadores de pessoas idosas com fragilidade clínica no domicílio.

## MÉTODOS

### Aspectos éticos

O estudo seguiu preceitos éticos e foi aprovado pelo comitê de ética UNIFESP. Os participantes foram esclarecidos sobre os objetivos da pesquisa por meio do Termo de Consentimento Livre e Esclarecido.

### Desenho, período e local do estudo

Estudo transversal de método misto, com estratégia sequencial explanatória, sendo a primeira etapa quantitativa - estudo observacional em epidemiologia norteado pela ferramenta STROBE; e a segunda etapa qualitativa - norteado pelo checklist COREQ^(14-16)^. Foi realizado em diferentes municípios do estado de São Paulo, no período de julho do ano de 2022 a abril de 2023^(14)^. Na etapa quantitativa, a coleta de dados ocorreu online pelo *Google Forms*. Na etapa qualitativa, os dados foram obtidos em entrevistas na plataforma *Google Meet* e, de forma presencial, no Centro de Assistência e Educação em Enfermagem da Universidade Federal de São Paulo (CAENF-UNIFESP).

### Amostra, critérios de inclusão e exclusão

A amostra foi composta por 170 familiares cuidadores principais acima de 18 anos que conviveram no mesmo domicílio, durante a pandemia COVID-19, em concordância com o cálculo amostral. Posteriormente, foram convidados aleatoriamente todos os participantes e 31 familiares concordaram em participar da etapa qualitativa a partir da sua disponibilidade de tempo.

Foram incluídos no estudo familiares de pessoas idosas com fragilidade clínica e que residiam no mesmo domicílio, acima de 18 anos. Neste estudo, foi considerada a pessoa idosa com fragilidade clínica com idade a partir de 65 anos que possui um ou mais sintomas característicos da síndrome. Foram excluídos os participantes que não se enquadram nesses critérios.

### Protocolo do estudo

Na etapa quantitativa, foi aplicado o Questionário Sociocontextual, elaborado pelas pesquisadoras, além dos instrumentos validados no Brasil: Escala Modos de Enfrentamento de Problemas (EMEP) para mensurar estratégias de enfrentamento em relação a estressores específicos, e o Questionário de Avaliação da Sobrecarga do Cuidador Informal (QASCI) para aferir a sobrecarga física, emocional e social do cuidador informal^(17,18)^.

Os dados da etapa qualitativa foram obtidos nas entrevistas semiestruturadas realizadas pelas pesquisadoras com expertise no tema. Antes da participação, os familiares foram esclarecidos sobre o estudo e dúvidas foram sanadas. Cada entrevista foi gravada em áudio com a autorização do familiar e teve duração de uma hora e iniciada com as seguintes perguntas norteadoras: “Como foi a experiência no cuidado ao seu familiar idoso com fragilidade clínica?”, “Quais estratégias de enfrentamento foram utilizadas no cuidado, na pandemia COVID-19?”.

### Análise dos resultados e estatística

Para as análises estatísticas, foi utilizado o *Statistical Package for the Social Sciences*, versão 21.0. Na correlação do escore dos domínios do EMEP com os domínios do QASCI, foi utilizado o Coeficiente de Correlação de *Spearman.* Para comparação dos escores dos domínios do EMEP e os domínios do QASCI com as variáveis Sociocontextuais categóricas, utilizaram-se o Teste de *Mann-Whitney* e o teste de *Kruskal-Wallis*, nível de significância de 5% (p-valor < 0,05). O cálculo do tamanho da amostra revelou o número 170 para a comparação entre os domínios do EMEP e a presença de demência na pessoa idosa, foi utilizado o tamanho amostral para “Tamanho Amostral para Diferença entre Médias”, nível de significância de 5% e um poder do teste de 80%.

As informações obtidas foram submetidas à análise de conteúdo no *Software MAXQDA.2024*, a partir da análise temática proposta por Bardin^(19)^. Os temas foram selecionados, e parte das narrativas foram codificadas para a criação de categorias. Após essa fase, foram realizadas as codificações axiais e seletivas resultando nas seguintes categorias: “Sobrecarga gerada pelo cuidado prestado”, “Estratégias de enfrentamento diante da situação de cuidar” e “Rede de apoio para promover o cuidado”.

## RESULTADOS

Na Tabela 1, nota-se a característica da amostra com dados sociocontextuais constituídos por 170 participantes, sendo 82% mulheres entre 50 e 69 anos (65,9%), 40% empregadas e 25,9% aposentadas; 68% possuem filhos, 31% com renda entre R$ 4.801 - 7.200,00. Quando questionadas sobre enfrentamento, 15,3% informaram não fazer nada para enfrentar, enquanto 15,3% informaram aceitação e 13,5% buscam por conhecimento. Quando questionadas sobre outras formas de lidar com as demandas de cuidado, 32,9% não utilizam outras formas e 14,7% responderam fé e gratidão.

**Tabela 1:** Características das variáveis sociocontextuais dos familiares cuidadores, Brasil, maio-dezembro/2022

Variáveis	Total
**Faixa etária**	
18-29	2 (1,2%)
30-49	44 (25,9%)
50-69	112 (65,9%)
70 ou mais	12 (7,1%)
Total de participantes	170
**Sexo**	
Feminino	140 (82,8%)
Masculino	29 (17,2%)
Total de participantes	169
Não informaram	*1*
**Quantas pessoas residem em sua casa?**	
2 pessoas	5 (2,9%)
3-4 pessoas	119 (70%)
5 ou mais pessoas	46(40,6%)
Total de participantes	170
**Têm filhos?**	
Sim	117 (68,8%)
Não	53 (31,2%)
Total de participantes	170

*Análise descritiva das variáveis categóricas. Cálculo da frequência e percentual.*

Quanto aos dados referentes ao familiar idoso, pode-se observar, na Tabela 2, que a faixa etária encontrada foi de 75 a 79 anos (27,1%) e de 80 a 84 anos (22,9%), 34,1% possuem de 5 a 6 sintomas, e 24,1% possuem de 7 a 8 sintomas, o que classifica fragilidade clínica, 43,5% moram há mais de cinco anos com a família e 22,9%, há três anos.

**Tabela 2:** Características das variáveis sociocontextuais referentes às pessoas idosas com fragilidade clínica, Brasil, maio-dezembro/2022

Variáveis	Total
**Qual a idade da pessoa idosa sob seus cuidados?**	
65-69	7 (4,1%)
70-74	17 (10%)
75-79	46 (27,1%)
80-84	39 (22,9%)
85-89	33 (19,4%)
90-94	22 (12,9%)
95-99	5 (2,9%)
Acima de 100	1 (0,6%)
Total de participantes	170
**Quantos sinais e sintomas ela possui?**	
1-2 sintomas	18 (10,6%)
3-4 sintomas	29 (17,1%)
5-6 sintomas	58 (34,1%)
7-8 sintomas	41 (24,1%)
9-10 sintomas	24 (14,1%)
Total de participantes	170
**Há quanto tempo a pessoa idosa mora com você? (em anos)**	
1	6 (3,5%)
2	19 (11,2%)
3	39 (22,9%)
4	32 (18,8%)
5	74 (43,5%)
Total de participantes	170
**Algum familiar auxilia você no cuidado?**	
Sim	96 (56,5%)
Não	74 (43,5%)
Total de participantes	170
**Qual é o parentesco?**	
Irmão/Cônjuge	54 (31,7%)
Pai/mãe	20 (11,8%)
Filho/neto	26 (15,3%)
Nenhum parente	70 (41,2%)
Total de participantes	170
**Você conta com a ajuda de um cuidador profissional?**	
Sim	72 (42,4%)
Não	98 (57,6%)
Total de participantes	170

*Análise descritiva das variáveis categóricas. Cálculo da frequência e percentual.*

Quando questionados sobre a rede de apoio, 56,5% dos participantes informaram possuir ajuda familiar no cuidado, sendo 31,7% ajudados por irmãos/cônjuges e 15,3% por filhos/netos, além de 57,6% não possuírem ajuda de cuidador profissional.

Na análise das escalas, observou-se que a sobrecarga emocional e financeira está presente em familiares cuidadores de pessoas idosas com diagnóstico de AVC, Parkinson e demências, ainda que possuam suporte familiar.

Quando correlacionada sobrecarga com estratégias de enfrentamento, conforme Tabela 3, notou-se que quanto maior a sobrecarga emocional e as implicações na vida pessoal causadas pelo cuidado prestado sem o auxílio da família, maior é a busca por práticas religiosas como estratégia de enfrentamento para o familiar cuidador principal. Todavia, há também maior busca por práticas religiosas de familiares cuidadores que possuem rede de apoio profissional, quando comparado com familiares cuidadores sem rede de apoio profissional.

**Tabela 3:** Características das variáveis da correlação dos domínios Escala Modos de Enfrentamento de Problemas por domínios Questionário de Avaliação da Sobrecarga do Cuidador Informal, Brasil, maio-dezembro/2022

		Enfrentamento focalizado no problema	Enfrentamento focalizado na emoção	Busca de práticas religiosas/pensamento fantasioso	Busca de suporte social
**Sobrecarga emocional**	R	0,07	0,23	0,21	0,01
	Valor de *p*	0,3919	**0,0026**	**0,0062**	0,8627
	N	170	170	170	170
**Implicações na vida pessoal do cuidador**	R	0,05	0,20	0,19	-0,04
	Valor de *p*	0,5572	**0,0099**	**0,0111**	0,5812
	N	170	170	170	170
**Sobrecarga financeira**	R	-0,03	0,00	0,08	-0,04
	Valor de *p*	0,6951	0,9659	0,2846	0,5834
	N	170	170	170	170
**Reações a exigências**	R	0,04	0,13	0,02	0,01
	Valor de *p*	0,6005	0,0987	0,8337	0,9349
	N	170	170	170	170
**Mecanismo de eficácia e de controle**	R	-0,04	0,00	-0,13	-0,13
	Valor de *p*	0,5764	0,9489	0,1016	0,0979
	N	170	170	170	170
**Suporte familiar**	R	0,00	0,18	0,12	-0,11
	Valor de *p*	0,9988	**0,0184**	0,1321	0,1470
	N	170	170	170	170
**Satisfação com o papel e com o familiar**	R	-0,18	-0,05	-0,12	-0,10
	Valor de *p*	**0,0223**	0,5513	0,1090	0,2070
	N	170	170	170	170

*R - Coeficiente de Correlação de Spearman.*

O familiar cuidador principal que tem suporte familiar e maiores implicações na sua vida pessoal apresenta maior enfrentamento focalizado na emoção. Enquanto o familiar cuidador principal que possui auxílio do cônjuge e com ausência de rede de apoio profissional apresenta menor enfrentamento focalizado no problema.

Familiares que residem com uma ou duas pessoas no mesmo domicílio sem rede de apoio familiar passam por maiores implicações na vida pessoal, comparando-se com os familiares que possuem auxílio de irmãos ou cônjuges, além de apresentarem maior enfrentamento focalizado no problema.

Familiares que moram com a pessoa idosa, há cinco anos ou mais, com idade entre 50 e 69 anos, apresentam menor suporte familiar, enquanto familiares que moram há três/quatro anos com a pessoa idosa e possuem entre 40 e 49 anos contam com o suporte familiar.

A partir da junção dos códigos das 31 entrevistas, a análise de conteúdo textual e teórica das narrativas gerou três categorias e subtemas que responderam aos objetivos propostos.

Partindo da categorização da análise textual para conferir aproximações possíveis e as semelhanças entre as categorias e subtemas da codificação aberta, utilizou-se a ferramenta *MaxMaps* do *software MAXQDA.2024* (Figura 1).

**Figura 1:** Mapa de categorias e subtemas, São Paulo, São Paulo, Brasil, 2023

Após a codificação aberta inicial, observaram-se as categorias e subtemas referentes às experiências do grupo focal avaliativo, sendo a primeira categoria referente à percepção dos familiares sobre sobrecarga gerada pelo cuidado prestado, exemplificada nas falas abaixo.

### Categoria 1: Sobrecarga gerada pelo cuidado prestado

Na primeira categoria, destacam-se as narrativas de alguns familiares com relatos sobre a rede de apoio familiar e profissional ineficaz, ocasionando aumento de sobrecarga, principalmente relacionada à pandemia da COVID-19.

*Me sinto muito sobrecarregada em cuidar dele, mesmo tendo ajuda* [...] *me sinto muito cansada!* (E.3)

*Me senti muito sobrecarregada, emocionalmente e fisicamente, de todas as maneiras, desde o período da pandemia que as coisas ficaram mais difíceis.* (E.8)

*Depois da pandemia, senti a sobrecarga emocional, acho que acabou somando tudo! Minha esposa e eu estamos sobrecarregados emocionalmente, fisicamente e financeiramente, não temos ninguém para contar além da cuidadora.* (E.27)

A presença da sobrecarga implicou em piora da saúde física e saúde mental do familiar cuidador destacada nas seguintes falas:

*Eu comecei a sentir dores por todo o corpo, sentia dor por tudo o que é lado do corpo, nas articulações, costas, em tudo!* (E.1)

*Para te falar a verdade, fiquei ainda mais cansada e estressada!* (E.20)

Outro destaque refere-se à presença de sobrecarga relacionada à inversão de papéis no sistema familiar, especialmente quando se passou a cuidar dos pais, o que remete ao luto antecipatório.

*Eu me senti emocionalmente sobrecarregada porque eu estava triste pela situação dela* [mãe]*, eu entendo que eu passei e estou passando por um luto de uma mãe independente.* (E.16)

*Em muitos momentos me senti muito sobrecarregada emocionalmente, ver minha mãe precisando de cuidados básicos mexeu muito comigo, não esperava ter que passar por isso com ela* [mãe]. (E.19)

*É um peso, uma sobrecarga indescritível, só quem passa sabe realmente como é ter que ver seus pais assim e ainda ter que cuidar, aprender na marra a cuidar. No final do dia, me sinto exausta, física e emocionalmente.* (E.30)

A segunda categoria refere-se às estratégias de enfrentamento desenvolvidas diante da situação e cuidar. As narrativas seguintes exemplificam algumas estratégias desenvolvidas.

### Categoria 2: Estratégias de enfrentamento diante da situação de cuidar

Na segunda categoria, os familiares relataram ter desenvolvido estratégias focadas na emoção e estratégias focadas no problema.

*Eu também enfrento, me descolando da situação, como se não fosse minha mãe e meu pai, como se fossem outras pessoas que eu estivesse cuidando. Às vezes eu me descolo, para não ficar tão pesado!* (E.7)

*r atrás de tudo o que ele tem direito pelo plano de saúde, levar eles aos médicos, ficar em cima dos cuidadores que vêm aqui para casa, são coisas que me dão muita segurança e tranquilidade* [...] *e saber que estou fazendo tudo o que eu posso mesmo.* (E.25)

*Depois que levei ela* [mãe] *ao médico, que as coisas começaram a mudar para mim, comecei a entender o que minha mãe realmente tinha e mudei minha forma de lidar com a situação, conversei muito com minha esposa também.* (E.27)

As práticas religiosas, a espiritualidade e a busca por conhecimento foram importantes estratégias de enfrentamento, conforme os relatos dos familiares.

*Não deixei de fazer as minhas orações, isso me fortalece muito, mesmo não indo na igreja mais.* (E.18)

*Eu pedia muito a Deus para me dar paciência, porque eu tenho pouca paciência.* (E.21)

*Eu buscava conhecimento para eu entender o que estava acontecendo e para poder cuidar da minha mãe da melhor forma possível.* (E.9)

A terceira categoria refere-se à rede de apoio para exercer o cuidado e elucida as características da rede como apoio familiar, apoio profissional e apoio ineficaz, exemplificadas nas falas abaixo.

### Categoria 3: Rede de apoio para promover o cuidado

Na terceira categoria, evidenciam-se os familiares cuidadores que possuem apenas rede de apoio profissional ou institucional, queixam-se por não poderem contar com rede de apoio familiar eficaz.

*Minha rede de apoio são só as Técnicas do Home Care e meus filhos financeiramente quando eu peço, mas não me ajudam o suficiente. Eu gostaria que eles fossem mais participativos, viessem mais em casa.* (E.3)

*Muito raramente meu irmão vinha me ajudar quando eu pedia, eram momentos bem pontuais. O Grupo Vida que me ajuda muito, as cuidadoras me ajudam.* (E.5)

*Como rede de apoio nós só temos a cuidadora, a família não nos ajuda em nada, nem em revezar ou cuidar.* (E.6)

Em relação às demandas de cuidado, os participantes identificam majoritariamente seus cônjuges e irmãos como rede de apoio familiar.

*Minhas irmãs me ajudaram muito nesse processo todo, somos em quatro irmãs e todas casadas, todas as famílias se ajudaram muito para cuidar dela* [mãe]*, ela* [mãe] *era o nosso maior foco!* (E.10)

*Meu marido é a única pessoa que me ajuda a cuidar do meu irmão quando ele está em casa.* (E.17)

*Minha rede de apoio sempre foi meu irmão.* (E.19)

*Minha irmã é a única pessoa que me ajuda a cuidar dele* [pai]*. Restante da minha família, os meus outros irmãos e parentes, não me ajudam em nada, eles nem aparecem na minha casa, não me ligam.* (E12)

## DISCUSSÃO

O envelhecimento populacional mundial está inserido nas mudanças sociodemográficas. Nesse cenário, maior expectativa de vida gera, como consequência, desenvolvimento de fragilidade clínica e de Doenças Crônicas Não Transmissíveis (DCNT) na população idosa, desencadeando maior necessidade de cuidado. Esses fatores acarretam aumento da demanda de cuidado e, por conseguinte, da sobrecarga emocional, física e financeira do cuidador familiar, principalmente das mulheres^(20,21)^.

Demonstrou-se que a maioria das cuidadoras familiares são mulheres (82,8%), pré-idosas e idosas jovens com idade entre 50 e 69 anos (65,9%), que contam com menor suporte familiar, quando comparado com familiares cuidadores com idade entre 30 e 49 anos. Esses dados corroboram o estudo que caracterizou o perfil de cuidadoras de pessoas idosas e comprova o fenômeno “feminização do cuidado”, isto é, mulheres como responsáveis pelo cuidado^(22)^.

Somado à questão cultural do cuidado familiar prestado por mulheres, outro fator importante está relacionado à morte prematura dos homens que, conforme indicadores do Ministério da Saúde, ocorre nas idades de 30 a 69 anos por DCNT, violência e outras. Esse dado é preocupante, pois, somente em 2023, no Brasil, foram constatados 111.544 óbitos causados por DCNT, sendo 55,7% de homens e 44,3% de mulheres. Esse cenário agrava a sobrecarga e o acúmulo dos papéis da mulher dentro do sistema familiar, além de implicar na qualidade do cuidado prestado^(23)^.

Os resultados qualitativos deste estudo confirmam a “feminização do cuidado”. Foi possível identificar, nas narrativas das cuidadoras familiares, a escassa participação dos homens nos cuidados prestados à pessoa idosa com fragilidade clínica, corroborando pesquisa qualitativa brasileira que observou a dedicação integral dos homens aos cuidados de um familiar idoso enquanto são solteiros, quando iniciam um relacionamento com uma mulher, ela passa a assumir a responsabilidade pelo cuidado familiar^(24)^. Na medida em que as cuidadoras familiares avançam na condição de pré-idosas e idosas jovens, podem desenvolver acúmulo de papéis e funções, fator que pode agravar a sua saúde.

Neste estudo, foi identificado que as cuidadoras participantes, no contexto da pandemia COVID-19, tiveram sua sobrecarga intensificada em razão do agravamento de saúde das pessoas idosas, majoritariamente de 75 a 84 anos, com fragilidade clínica, sob seus cuidados, apresentando de cinco a oito sintomas classificatórios somados aos diagnósticos de AVC, Parkinson, demências e outras DCNT, pois houve o comprometimento do atendimento à saúde na rede pública e redução na prestação de cuidados na rede privada, em virtude da necessidade do isolamento social.

Esses achados convergem para pesquisas que concluíram que pessoas idosas, com ou sem demência, tiveram alterações clínicas durante a pandemia, em razão do isolamento social, inatividade física forçada e restrição de estímulos externos, tendo também comprometimento cognitivo^(25,26)^. Nesse aspecto, o agravamento de saúde da pessoa idosa dependente intensificou a demanda e a carga de cuidado, implicando no aumento de sobrecarga física, emocional e financeira, pois, além do isolamento pandêmico, essas cuidadoras também tiveram acompanhamento de saúde precário.

Os dados obtidos revelam menor suporte familiar no cuidado quando o integrante idoso mora no mesmo domicílio há cinco anos ou mais, comparando-se com o tempo de três/quatro anos em que há maior suporte. Em concordância, pesquisa transversal realizada com 348 pessoas idosas que moram com seus familiares evidenciou que, de acordo com os anos passados e o avanço da dependência de cuidados, há redução da rede de apoio familiar ao familiar cuidador. Dessa forma, percebe-se que, nesse cenário, faz-se necessária a presença da enfermagem como apoio profissional para a atenção às pessoas idosas dependentes e aos familiares cuidadores^(27)^.

Outros aspectos evidenciados nas narrativas das cuidadoras foram as implicações e/ou repercussões na vida pessoal como piora da saúde física. Algumas relataram o surgimento de problemas de coluna, fadiga e de saúde mental, como estresse e ansiedade relacionados à ausência de rede de apoio familiar. Esses fatores também foram encontrados em estudo realizado sob coordenação da Fundação Oswaldo Cruz, demonstrando que conviver com uma pessoa idosa dependente de cuidado durante a pandemia levou à piora da saúde, do estado de ânimo e piora do sono^(28)^. Ressalta-se que a piora na saúde da cuidadora familiar pode impactar a condição do cuidado prestado. Para minimizar essa situação, necessário se faz que a equipe de saúde estenda seu cuidado para a família do paciente idoso.

Diante da prestação de cuidados e sobrecarga, as cuidadoras familiares desenvolvem estratégias focalizadas na emoção ou no problema. Neste estudo, destaca-se a busca por práticas religiosas e pela espiritualidade como importante estratégia de superação da adversidade, além da busca por conhecimento por meio de leitura e discussão com profissionais de saúde e rede virtual social.

Em concordância, pesquisa com método misto que investigou a espiritualidade/religiosidade como estratégia de enfrentamento no contexto da pandemia COVID-19 concluiu que a espiritualidade/religiosidade, quando estimuladas, contribuem para minimizar danos adversos à saúde mental^(29)^. Observa-se que a dimensão espiritual é capaz de contribuir para a melhora do bem-estar físico e emocional, entretanto, ressalta-se que outras estratégias, como convívio social, prática de atividade física e de lazer, busca por atendimento especializado, terapia individual e familiar, terapias integrativas e complementares, são essenciais para o autocuidado da cuidadora familiar^(30)^.

No que tange às estratégias de enfrentamento e cuidado, o apoio familiar e profissional é crucial na promoção da saúde e do bem-estar. Nos dados qualitativos, foi relatado o aumento da sobrecarga da cuidadora pré-idosa e idosa jovem em razão da ineficácia da rede de apoio familiar (majoritariamente formada por irmãos e cônjuges), inclusive em situações em que existe a rede de apoio profissional. Em concordância, pesquisa qualitativa realizada concluiu que as idosas cuidadoras possuíam diferentes redes de apoio eficazes que minimizam a sobrecarga do cuidado prestado^(31)^. Portanto, percebe-se que a rede de apoio eficaz é uma das estratégias de enfrentamento que reduzem a sobrecarga, dentre elas, destaca-se o auxílio da equipe de enfermagem na promoção do cuidado estendido para a família.

A enfermagem como componente da rede de apoio profissional pode proporcionar educação em saúde e planejar plano de cuidado para a família, de acordo com as demandas da pessoa idosa com fragilidade clínica e da cuidadora familiar principal. Nas entrevistas deste estudo, ficou evidente a importância da rede de apoio profissional da equipe de enfermagem, atuando com base na teoria sistêmica.

A Teoria dos Sistemas convida a enfermagem a promover o cuidado integral e sistêmico, que contempla a pessoas idosa e sua cuidadora familiar principal considerando a saúde um processo dinâmico. Nessa perspectiva, a equipe de saúde, inclusive a de enfermagem, necessita ampliar seu olhar centrado no paciente para a família, auxiliar a família na identificação e elaboração da rede de apoio e colaborar para a elaboração de estratégias de enfrentamento^(32,33)^. Além disso, a enfermagem pode colaborar na promoção da autonomia do funcionamento do sistema familiar, auxiliando na identificação de seus pontos fortes e no desenvolvimento de seus próprios recursos. Pesquisa quantitativa feita em uma cidade mineira corrobora o estudo em questão ao concluir que a enfermagem contribui para o auxílio da saúde da família^(34)^.

### Limitações do estudo

Como limitação neste estudo, destaca-se ser apenas um recorte regional para o estado de São Paulo, com um número de participantes que não representa a totalidade regional e nacional. Os dados foram coletados no contexto da pandemia da COVID-19, sendo um obstáculo para maior abrangência. Ressalta-se que, embora o cenário atual seja pós-pandêmico, as famílias seguem sobrecarregadas pelas demandas de cuidado potencializadas pelas necessidades de cuidados agravadas. Diante do exposto, ressalta-se a importância de investimentos em inéditas pesquisas para propor e avaliar intervenções para o alívio da sobrecarga e auxílio do desenvolvimento de estratégias para o familiar cuidador principal pré-idoso e idoso jovem no cuidado à pessoa idosa dependente.

### Contribuições para a área da Enfermagem

Este estudo traz contribuições significativas para a área da enfermagem, evidenciando a importância de ampliar o cuidado aos familiares dos pacientes idosos, ou seja, ampliar as estratégias de cuidado para as famílias. Em vista disso, torna-se relevante e urgente estabelecer planejamento de ações de cuidados e intervenções personalizadas para as famílias conforme suas demandas específicas, visto que uma pré-idosa ou idosa jovem cuidando de outras pessoas idosas mais velhas também precisa de cuidados em saúde.

## CONCLUSÕES

O fenômeno de ter uma pessoa idosa com fragilidade clínica no domicílio tem aumentado mundialmente, gerando modificações na estrutura e dinâmica da família, especialmente no que se refere à cuidadora familiar principal pré-idosa e idosa jovem. Esse estudo evidencia que o aumento da sobrecarga dos familiares cuidadores impacta a rotina do cuidado, sendo a rede de apoio familiar e social uma estratégia de enfrentamento eficaz bem como a busca por práticas religiosas, pela espiritualidade e por conhecimento por meio da leitura e discussão com profissionais de saúde e rede virtual social.

Destaca-se a importância de atenção para a saúde da cuidadora familiar idosa que pode adoecer com a sobrecarga somada às suas demandas clínicas. Diante disso, é imprescindível que as equipes de saúde ampliem sua atuação para a família da pessoa idosa em recomendação ao objetivo 3, item 3.4, agenda 2030 - ONU dos Objetivos de Desenvolvimento Sustentável (ODS).

## Data Availability

Os dados da pesquisa estão disponíveis no corpo do artigo.
